# Clinicians’ Trust in AI-Based Clinical Recommendations Across Controlled Primary Care–Style Clinical Vignettes: Nurse-Dominant Experimental Pilot Study

**DOI:** 10.2196/97649

**Published:** 2026-10-08

**Authors:** Avishek Choudhury, Yeganeh Shahsavar, Ayse P Gurses

**Affiliations:** 1 Industrial and Management Systems Engineering Benjamin M Statler College of Engineering and Mineral Resources West Virginia University Morgantown, WV United States; 2 Armstrong Institute Center for Health Care Human Factors Johns Hopkins Medicine Johns Hopkins University Baltimore, MD United States; 3 Department of Health Policy and Management Johns Hopkins Bloomberg School of Public Health Johns Hopkins University Baltimore, MD United States

**Keywords:** artificial intelligence, clinical decision support systems, trust composite, human-AI interaction, trust calibration, behavioral reliance, registered nurses, clinical vignettes, perceived difficulty, repeated interaction

## Abstract

**Background:**

Safe integration of AI-enabled clinical decision support requires understanding whether users’ trust and behavioral reliance are appropriately calibrated to recommendation quality.

**Objective:**

This pilot study examined a 3-item vignette-level trust construct and accept or reject behavior across sequential primary care–style clinical vignettes.

**Methods:**

A total of 68 health care professionals, including 59 (86.8%) registered nurses and 9 (13.2%) physicians, each evaluated 21 clinical vignettes from 1 of 2 series. Each series contained 10 correct and 11 intentionally incorrect AI recommendations. Participants accepted or rejected each recommendation and responded to 3 questionnaire items measuring trust, perceived transparency, and likelihood of acting on the recommendation before receiving correctness feedback and points. The unrounded item mean formed the trust composite. In the behavioral analysis, 50 unrecorded accept or reject responses were classified as rejections. A pooled cross-classified linear mixed-effects model was constructed to assess associations between the trust composite, recommendation correctness, vignette position, and participant and vignette characteristics. It included random intercepts for participant and vignette item, with case series included as a nuisance adjustment.

**Results:**

Participants accepted 374 of 748 (50%) incorrect recommendations and rejected 112 of 680 (16.47%) correct recommendations. Incorrect recommendations received lower prefeedback trust than correct recommendations (β=−0.772, 95% CI −1.036 to −0.507; standardized effect=−0.419). Trust declined modestly across vignette positions (β=−0.027, 95% CI −0.048 to −0.005; standardized effect=−0.088), with a decline in case series A but not case series B. Baseline intention to use AI was positively associated with trust (β=0.391, 95% CI 0.110-0.672; standardized effect=0.243), whereas higher perceived diagnostic difficulty was negatively associated with trust (β=−0.474, 95% CI −0.648 to −0.301; standardized effect=−0.257). In the exploratory lagged model, trust in the preceding recommendation was associated with trust in the current recommendation (β=0.266, 95% CI 0.210-0.322; standardized effect=0.264).

**Conclusions:**

Self-reported trust differentiated correct from incorrect recommendations in aggregate, while acceptance and rejection were not fully aligned with recommendation correctness. These preliminary findings identify a potential evaluation problem: lower trust in incorrect advice does not necessarily imply that users will reject it. The findings do not establish real-world clinical effects.

## Introduction

AI-enabled clinical decision support systems (CDSS) have the potential to improve diagnostic and treatment workflows across health care settings by augmenting clinicians’ productivity, particularly in environments characterized by workload and uncertainty [1-3]. Yet, the adoption of AI at the bedside has been slower than expected [4]. One major reason for this slow uptake is clinicians’ trust in AI systems [5]. Clinicians must feel confident that AI recommendations are reliable and safe before they are willing to incorporate them into their clinical workflow. However, integrating AI into health care without fully understanding how clinicians interact with these systems and how their trust develops over time can create risks for patient safety [6,7].

AI performance may fluctuate depending on the clinical scenario, data quality, or system limitations [8]. If clinicians overtrust AI recommendations, they may accept incorrect advice, while undertrust may lead them to ignore useful guidance. Both situations can negatively affect patient care. Therefore, to safely and effectively integrate AI into clinical practice, it is essential to understand how clinicians develop, adjust, and act on trust in AI systems, as well as the factors that influence this trust [6,9]. In many ways, understanding these trust dynamics is just as important as improving the technical performance of AI algorithms themselves [10].

In clinical environments, which are high-paced and high-stakes work systems, maintaining appropriate trust in AI systems is critical. Health care workers (nurses and physicians) often need to make rapid decisions while managing multiple sources of information, and in such settings, clinicians must quickly determine when to accept, question, or override AI recommendations. While they commonly adopt a trust-but-verify approach when working with residents or colleagues, AI systems differ in that they lack shared accountability and interactive clarification. Trust in AI can be confounded with a user’s self-confidence [11,12]. If a user lacks self-confidence, they may report lower trust in the AI not because the AI is unreliable, but because they doubt their own capacity to use it safely. Distinguishing self-confidence from trust is necessary to identify the right remedies (improve model explanations vs improve user training). Additionally, positive past performance may gradually increase trust in AI recommendations [13]. At the same time, as trust grows, users may become less likely to critically scrutinize AI outputs, allowing erroneous recommendations to pass through the decision-making process without sufficient evaluation. Conversely, negative past experiences with AI may make users more skeptical of the system, reducing their willingness to rely on AI recommendations even when they are accurate [5,7,14-16]. These dynamics highlight the importance of understanding how nurses’ and physicians’ trust evolve through repeated interactions with AI systems. Although AI decision support tools often perform with high accuracy, infrequent errors can hinder trust in the technology.

Real-world clinical work is demanding, and trust in automation is known to vary with workload, uncertainty, and perceived task difficulty [17-19]. A growing literature has examined global attitudes toward AI in health care, yet far fewer studies have explored how trust in AI can change in a clinical setting [10,20]. Existing work has largely focused on isolated psychological or demographic predictors such as prior trust, confidence, or experience without simultaneously examining how these individual characteristics interact with contextual pressures such as time constraints and perceived diagnostic complexity [21,22].

Building on trust calibration and human-automation reliance frameworks, we conceptualized trust as a dynamic evaluation that should ideally correspond to the perceived trustworthiness and observed reliability of the AI system. Foundational trust-in-automation theory argues that trust guides reliance when users cannot fully inspect or understand an automated system, and that safe human-automation interaction requires “appropriate reliance” rather than either blind acceptance or blanket rejection of automation [23,24]. Automation-reliance research further distinguishes subjective trust from observable patterns of use, misuse, disuse, and overreliance, emphasizing that users may behave in ways that do not perfectly match their stated trust. This distinction is especially important in clinical decision support, where automation bias can occur when clinicians overrely on CDSS recommendations and reduce independent information seeking or verification [25]. Recent health care AI studies and reviews similarly emphasize that clinicians’ trust in AI-CDSS is shaped by clinical reliability, transparency, validation, usability, prior experience, and professional expertise, and that trust should be examined separately from behavioral reliance on AI advice [16,26,27]. Therefore, appropriate reliance occurs when clinicians accept useful or correct AI recommendations and question or reject recommendations that are unsafe, incorrect, or insufficiently supported; overreliance and underreliance represent mismatches between AI reliability and clinician behavior. In repeated AI-assisted decisions, trust may be influenced by dispositional attitudes toward AI, the visible plausibility of each recommendation, feedback from prior clinical vignettes, and task conditions such as difficulty and time constraints [13]. Therefore, this pilot study distinguished reported trust-related evaluation from behavioral acceptance of AI recommendations.

This experimental pilot study had 3 aims. The primary aim was to characterize how the vignette-level trust composite changed across 21 sequential AI-assisted clinical vignettes and how it was associated with recommendation correctness, vignette position, baseline AI attitudes, participant characteristics, and perceived diagnostic difficulty. The second aim was to describe whether behavioral reliance, measured by accepting or rejecting recommendations, aligned with programmed correctness. The exploratory aim was to examine the association between trust in consecutive recommendations.

## Methods

### Study Design

This online experimental pilot study used repeated observations across 2 fixed series of 21 clinical vignettes. For each clinical vignette, participants reviewed a fictional patient presentation and a large language model (LLM)–generated diagnosis and recommendation, indicated whether they accepted or rejected the recommendation, and rated their trust. The same response process was repeated sequentially for all 21 clinical vignettes in the assigned series. No real patient encounters or patient outcomes were evaluated.

### Clinical Vignettes

Before generating any study materials, the authors provided Current Medical Diagnosis and Treatment 2010 [28] to ChatGPT (GPT-4; OpenAI) as reference material. ChatGPT was then used to draft short, fictional clinical vignettes and their associated diagnoses and recommendations. The clinical vignettes concerned common adult presentations intended to be understandable without subspecialty knowledge. They were not drawn from real patient records. The generation tasks involved creating the patient descriptions and associated reference diagnoses and recommendations, with intentionally incorrect participant-facing alternatives included where required by the experimental design.

The authors reviewed the generated materials before expert physician review. They compared the initial diagnoses with the textbook and checked the clinical vignettes and recommendations for readability, completeness, clinical coherence, and internal consistency. A physician subsequently conducted the expert physician review, assessing the plausibility of each presentation, the compatibility of the proposed diagnosis with the clinical information, and the consistency of each participant-facing recommendation with its intended correct or incorrect status. She finalized the reference diagnoses and approved the materials for use in the experiment. No corrections to the initial reference diagnoses were required during either the authors’ textbook-based review or the physician’s review.

The absence of diagnosis-level corrections should be distinguished from the deliberately incorrect recommendations used in the experiment. Reference diagnoses established what was considered correct for each clinical vignette; participant-facing recommendations could intentionally differ from those diagnoses to implement the planned correctness sequence. These incorrect recommendations were experimental stimuli, not diagnostic errors left unaddressed during review.

Multimedia Appendix 1 describes the production and review procedure and provides prompt templates for reference-guided vignette generation, diagnosis and recommendation generation, and intentionally incorrect alternatives.

### Participant Recruitment

Participants were recruited between August and September 2025 through Centiment, a paid online audience-panel service [29]. The survey was distributed to the provider’s health care worker panel. Eligibility was based on self-reported professional designation as a registered nurse (RN) or physician and reported clinical experience. RNs were included because they are important users of digital clinical information and decision support and routinely contribute to assessment, monitoring, triage, escalation, and care coordination [21,22]. The clinical-vignette task examined evaluation of AI recommendations and reliance behavior; it was not intended to test participants’ independent diagnostic or prescribing competence.

No independent credential, licensure, employer, or National Provider Identifier verification was performed. Centiment applies proprietary quality procedures, including duplicate and automated-response detection, device or browser fingerprinting, ReCAPTCHA, and fraud scoring. These procedures were performed by the panel provider; the research team did not receive IP addresses or device identifiers. The number invited, prescreened, or excluded by the provider and the numerical fraud-score threshold were not available to the research team.

### Study Procedures

The 42 clinical vignettes were allocated to two 21-vignette series of broadly comparable scope and expected difficulty, without formal matching on independent prestudy difficulty ratings. Each series included 10 correct and 11 intentionally incorrect recommendations, giving a programmed accuracy rate of 47.62%. Both began with 4 correct recommendations followed by 2 incorrect recommendations; later correctness positions differed. Figure 1 shows the series-specific sequences. Check marks (✓) indicate recommendations classified as correct; cross marks (x) indicate intentionally incorrect recommendations. The two case series shared the same status through Trial 8 but differed at selected later trial positions. Accept or reject decisions and ratings were submitted before correctness feedback, and points were displayed for each trial. Participants rated the perceived difficulty of every clinical vignette so that variation in subjective difficulty could be described and included in the analysis.

**Figure 1:**
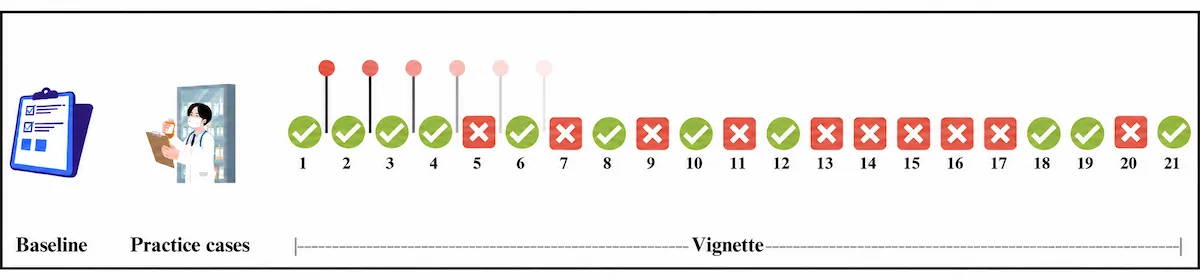
Actual programmed sequence of AI recommendation correctness across the 21 analyzed cases.

Participants completed the study in one Qualtrics session and were randomly assigned to case series A or B. The session included electronic consent, task instructions, baseline questions, a demonstration vignette, and the 21 analyzed clinical vignettes. A programmed attention-check screen appeared between the fifth and sixth analyzed clinical vignettes. The demonstration vignette and attention-check screen were excluded from the analyses. Case series A had no fixed response window; case series B used a 45-second response window.

On each clinical-vignette screen, participants reviewed the patient presentation and AI recommendation, indicated whether they accepted or rejected the recommendation, and responded to 3 trust questionnaire items and a perceived-difficulty question. The accept or reject item preceded the trust questions to prioritize eliciting behavioral reliance before explicitly asking participants to reflect on trust. All responses appeared on the same screen and were submitted before correctness feedback or points were displayed.

After submission, participants received feedback about the programmed correctness of the AI recommendation. They received +10 points for accepting a correct recommendation or rejecting an incorrect recommendation and −10 points for the opposite decisions. Feedback and scores from preceding clinical vignettes were intentionally available to inform responses to subsequent recommendations. In case series B, the page advanced when the 45-second response window expired; the analytic handling of unrecorded decisions is specified below under the “Analysis” section.

### Outcome Measurement

The primary outcome was vignette-level trust in the AI recommendation. Trust was operationalized through 3 investigator-developed questionnaire items addressing perceived trust, perceived transparency, and likelihood of acting on the recommendation. These items were specified as indicators of the study’s trust construct rather than as 3 separate primary outcomes; their unrounded mean was the trust composite. The conceptual framing drew on trust-in-automation literature [23,24]. The observed accept or reject response was a separate behavioral-reliance outcome. Baseline measures are summarized in Table 1, and the clinical-vignette measures are given in Table 2.

**Table 1:** Baseline participant measures.

Construct	Survey items	Scale or scoring
Professional designation	What is your professional or academic background?	Registered nurse Physician
Clinical experience	How many years of experience do you have in your current field or line of work?	<1 year 1-2 years 3-5 years 6-10 years >10 years
Prior AI use	In the last 6 months, have you ever used AI in medical practice?	Yes No
Baseline trust in AI (3-item composite)	General trust in AI, perceived transparency of AI recommendations, and likelihood of acting on an AI recommendation.	Each item: 1=none at all to 7=completely Composite=unrounded mean of available items (minimum 2 of 3)
Intent to use AI	I would like to use AI in my clinical practice.	1=strongly disagree to 5=strongly agree
Self-confidence	Nine retained items: I handle new situations with relative comfort and ease; I feel positive and energized about life; I keep trying even after others have given up; If I work hard to solve a problem, I will find the answer; I achieve the goals I set for myself; people give me positive feedback on my work and achievements; when I overcome an obstacle, I think about the lessons I have learned; I believe that if I work hard, I will achieve my goals; I have contact with people with similar skills and experience whom I consider successful.	Each item: 1=not at all to 5=very often Mean of 9 retained items

**Table 2:** Clinical-vignette measures completed before correctness feedback.

Construct	Survey item	Scale
Perceived diagnostic difficulty	How difficult do you think it would be to diagnose this patient case based on the information provided without AI assistance?	1=very easy to 5=very difficult
Vignette-level trust in the AI recommendation (3-item composite)	How much do you trust the AI-based recommendation? How transparent do you find the AI-based recommendation? How likely are you to act upon the AI-based recommendation?	Each item: 1=none at all to 7=completely Composite=unrounded mean of the 3 items

Separate 1-factor confirmatory factor analysis (CFA) summaries based on polychoric correlations were aligned to the 21 analyzed clinical-vignette positions. Average variance extracted (AVE) ranged from 0.76 to 0.92, coefficient omega from 0.90 to 0.97, and Cronbach α from 0.87 to 0.96. The 3 items showed strong internal consistency; these results do not make the self-reported composite equivalent to observed acceptance or rejection. Vignette-specific factor loadings and reliability estimates are reported in Multimedia Appendix 2.

Baseline trust was calculated as the unrounded mean of the corresponding 3 baseline items, using at least 2 available responses, as specified in Table 1. Cronbach α was 0.938 among participants with complete baseline items. Self-confidence was the unrounded mean of the 9 retained 1-5 items in Table 1, informed by self-efficacy theory [30]. The supplied self-confidence CFA reported AVE=0.65, coefficient Ω=0.94, and Cronbach α=0.91 (Multimedia Appendix 2).

### Data Collection

Before the clinical-vignette sequence, participants reported their professional designation, clinical experience, prior AI use, baseline trust, intention to use AI, and self-confidence. During each clinical vignette, the accept or reject decision was elicited as a binary choice, the three trust items as 1-7 ratings, and perceived diagnostic difficulty as a 1-5 rating. Responses were linked by anonymous study record, case series, and clinical-vignette position for repeated-measures analysis.

The first 20 eligible participants formed a pilot test to assess survey access, clarity of instructions, screen functioning, and whether participants could understand and answer the questions. They met the same eligibility criteria and completed the same consent process, measures, clinical-vignette sequence, and feedback procedure as the remaining participants. No technical or comprehension problems were identified, and no changes were made; these responses were retained in the final analytic sample.

### Analysis

The hypotheses concerned the trust composite: H1a predicted a difference between correct and intentionally incorrect recommendations after accounting for vignette position and participant and vignette variability; H1b predicted a positive association between trust in consecutive recommendations; H2 predicted higher trust among participants with stronger baseline intention to use AI; and H3 predicted lower trust for clinical vignettes perceived as more difficult.

The demonstration vignette and attention-check screen were excluded. All 3 trust items and perceived-difficulty responses were complete for the 68 analyzed participants across 21 clinical vignettes, yielding 1428 participant-vignette observations. The data were organized in long format. The 5-point difficulty rating was categorized as lower difficulty (very easy or easy) or higher difficulty (moderate, difficult, or very difficult); a sensitivity model retained the original 1-5 score. Perceived difficulty was treated as a concurrent subjective task-context predictor rather than as a preexposure causal confounder.

Recommendation correctness was coded separately for each case series using the programmed status and cross-checked against Qualtrics task-score fields. Clinical-vignette position was centered at position 11. Correctness remained partly linked to vignette content, position, and the fixed reliability sequence; coefficients involving correctness were therefore interpreted as adjusted associations rather than causal effects.

The primary analysis pooled all 68 participants. A parsimonious cross-classified linear mixed-effects model assessed associations with the trust composite and included random intercepts for participant and vignette item, with 42 distinct vignette items. Fixed effects were recommendation correctness, centered clinical-vignette position, case series, professional designation, baseline trust, intention to use AI, self-confidence, clinical experience (≤10 vs >10 years), and perceived difficulty. Continuous participant-level predictors were mean-centered. Case series was a nuisance adjustment because vignette content and response window varied together; between-series differences were not attributed to response timing.

The primary model was fitted separately within each case series, and a pooled vignette position × case series model examined differences in sequence slopes. A secondary interaction model examined whether the incorrect-versus-correct trust difference varied with vignette position, baseline trust, intention to use AI, self-confidence, experience, or perceived difficulty. These interactions were excluded from the parsimonious primary model and interpreted as hypothesis-generating. An exploratory lagged model used positions 2-21 to relate trust in the current recommendation to trust in the immediately preceding recommendation while adjusting for current correctness, vignette position, case series, difficulty, and participant variables. Models with and without preceding-vignette trust were compared by maximum likelihood; the lagged coefficient was interpreted as a temporal association rather than evidence of a causal mechanism.

The study and analyses were not preregistered. Models were estimated by restricted maximum likelihood in Python 3.13 (Python Software Foundation) using *statsmodels* (version 0.14.6). Maximum likelihood was used for the lagged-model comparison. All reported models converged. Estimates were summarized using coefficients, SEs, 2-sided Wald tests, and 95% CIs. Standardized effects came from parallel models with z-standardized outcomes and continuous predictors, retaining the original categorical coding. Marginal *R*² described variance explained by fixed effects; conditional *R*² included fixed and random effects. Trust-composite end point frequencies were also examined.

Behavioral reliance was summarized by acceptance and rejection relative to programmed recommendation correctness. A total of 50 case series B observations had no recorded accept or reject response. For the primary descriptive analysis, these observations were assigned to the rejection category under the assumption that participants who trusted a recommendation sufficiently to act on it would record acceptance within the 45-second response window. This rule retained all presented clinical vignettes and treated unrecorded decisions as nonacceptance; it did not establish that participants actively rejected or distrusted the recommendation. A descriptive sensitivity summary excluded unrecorded decisions instead of recoding them; the resulting counts and denominators are reported in Multimedia Appendix 3.

### Ethical Considerations

The West Virginia University (WVU) Institutional Review Board (IRB) approved the study under the WVU Flexibility Review Model (protocol number 2502117081). All participants provided electronic informed consent before beginning the survey. The study did not request names, contact information, IP addresses, licensure identifiers, employer information, or other direct identifiers; the analytic dataset contained anonymous responses. Study files were stored in password-protected locations accessible only to authorized research personnel. Participants were recruited and compensated through Centiment according to the provider’s panel procedures; the exact participant payment was not available to the research team.

This was an online clinical-vignette experiment without real patient encounters or patient outcomes. Applicable CONSORT-AI (Consolidated Standards of Reporting Trials–Artificial Intelligence) reporting principles informed descriptions of eligibility, allocation, participant flow, recommendation presentation, human-AI interaction, and analysis [31].

## Results

### Participant Flow and Characteristics

A total of 113 individuals participated in the survey. Sixty-eight met the eligibility and completeness criteria and were included in the analysis. The panel provider did not supply the number invited or prescreened, the number excluded through provider-level fraud or quality procedures, or sufficient information to distinguish ineligible from incomplete records among the remaining 45 participants. The final sample included 59/68 (86.76%) RNs and 9/68 (13.24%) physicians, with 34 participants assigned to each case series. All 68 analyzed participants provided complete responses to the 3 trust questionnaire items and the perceived-difficulty item across 21 clinical vignettes.

Baseline descriptive statistics, reported as means and SDs, are presented in Table 3. The mean baseline trust composite was 4.37 (SD 1.64); mean intention to use AI was 3.35 (SD 1.16); and mean self-confidence was 4.02 (SD 0.70).

**Table 3:** Participant characteristics and baseline measures^a^.

Characteristic		Case series A (n=34)	Case series B (n=34)	Total (n=68)
Professional designation, n (%)				
	Registered nurse	31 (91.18)	28 (82.35)	59 (86.76)
	Physician	3 (8.82)	6 (17.65)	9 (13.24)
Experience (years), n (%)				
	<1	2 (5.88)	0 (0.00)	2 (2.94)
	1-2	1 (2.94)	3 (8.82)	4 (5.88)
	3-5	8 (23.53)	2 (5.88)	10 (14.71)
	6-10	10 (29.41)	4 (11.76)	14 (20.59)
	>10	13 (38.24)	25 (73.53)	38 (55.88)
Prior AI use, n (%)				
	Yes	16 (47.06)	16 (47.06)	32 (47.06)
	No	18 (52.94)	18 (52.94)	36 (52.94)
Baseline trust composite, mean (SD)		4.28 (1.66)	4.46 (1.64)	4.37 (1.64)
Intent to use AI, mean (SD)		3.41 (1.08)	3.29 (1.24)	3.35 (1.16)
Self-confidence, mean (SD)		3.95 (0.66)	4.09 (0.74)	4.02 (0.70)

^a^The baseline trust composite is the unrounded mean of general trust, perceived transparency, and likelihood-to-act items. Self-confidence is the unrounded mean of 9 retained items.

### Perceived Diagnostic Difficulty

Of the 714 difficulty ratings in each series, 342 (47.90%) were categorized as higher difficulty in case series A and 355 (49.72%) in case series B. The position-specific distributions are shown in Multimedia Appendix 3.

### Trust Across Clinical Vignettes

The trust composite varied across the 21 clinical vignettes in both series (Figure S2 in Multimedia Appendix 3). Case series A increased from a mean of 5.04 at position 1 to 5.38 at position 4, decreased to 3.56 at the first incorrect recommendation at position 5, and ended at 3.21. Case series B began at 4.80, decreased to 3.54 at position 5, and ended at 4.32. Grouped means by case series, difficulty, and correctness are shown in Multimedia Appendix 3.

Across 1428 trust composites, 123 (8.61%) were at the minimum of 1 and 177 (12.39%) were at the maximum of 7; 300 scores (21.01%) occurred at either end point. Scores of 2 or lower accounted for 17.72%, and scores of 6 or higher accounted for 27.03%.

### Behavioral Reliance

Across both series, 374 of 748 (50%) incorrect recommendations were accepted, and 112 of 680 (16.47%) correct recommendations were in the rejection category. Each series contributed 714 decision instances: 374 with incorrect and 340 with correct recommendations. Incorrect recommendations were accepted 185 times in case series A (49.47% of incorrect recommendations; 25.91% of all series A observations) and 189 times in case series B (50.53%; 26.47% of all series B observations). Correct recommendations were in the rejection category 52 times in case series A (15.29% of correct recommendations; 7.28% of all series A observations) and 60 times in case series B (17.65%; 8.40%).

At clinical vignette 5, the first incorrect recommendation in both series, 16 participants in case series A accepted and 18 rejected the recommendation; in case series B, 12 accepted and 22 responses were classified in the rejection category. Acceptance was above zero for every incorrect recommendation. When both series returned to a correct recommendation at position 17, 25 participants in case series A and 28 in case series B accepted it. Position-specific counts are shown in Multimedia Appendix 3.

Of the 50 unrecorded case series B decisions, 26 occurred for correct and 24 for incorrect recommendations. When only recorded responses were counted, incorrect recommendations were accepted in 374 of 724 (51.66%) observations, and correct recommendations were explicitly rejected in 86 of 654 (13.15%) observations. In case series B alone, explicit rejection of correct recommendations was 34 of 314 (10.83%) recorded responses, compared with 60 of 340 (17.65%) under rejection-category coding. Complete denominators for both summaries are provided in Multimedia Appendix 3.

### Primary Mixed-Effects Model

Table 4 presents the pooled parsimonious cross-classified model. Incorrect AI recommendations were associated with lower prefeedback trust than correct recommendations (β=−0.772, 95% CI −1.036 to −0.507; standardized effect=−0.419). Trust also declined modestly across clinical vignettes (β=−0.027, 95% CI −0.048 to −0.005; standardized effect=−0.088). Intention to use AI was positively associated with trust (β=0.391, 95% CI 0.110-0.672; standardized effect=0.243), whereas higher perceived difficulty was negatively associated with trust (β=−0.474, 95% CI −0.648 to −0.301; standardized effect=−0.257). CIs for the remaining fixed effects included zero. The sensitivity model retaining the original 1-5 difficulty score yielded the same substantive pattern; each 1-point increase in difficulty was associated with a 0.404-point lower trust composite (95% CI −0.495 to −0.312).

**Table 4:** Parsimonious cross-classified mixed-effects model predicting the vignette-level trust composite^a^.

Predictor	β (SE)	95% CI	*P*	Standardized effect
Intercept	4.795 (0.249)	4.307 to 5.283	<.001	—^b^
Vignette position (centered)	−0.027 (0.011)	−0.048 to −0.005	.02	−0.088
AI recommendation incorrect	−0.772 (0.135)	−1.036 to −0.507	<.001	−0.419
Case series B vs A (nuisance adjustment)	0.174 (0.300)	−0.414 to 0.762	.56	0.094
Professional designation (physician)	0.243 (0.401)	−0.542 to 1.029	.54	0.132
Baseline trust composite	0.072 (0.095)	−0.114 to 0.259	.45	0.064
Intent to use AI	0.391 (0.143)	0.110 to 0.672	.006	0.243
Self-confidence	0.047 (0.198)	−0.341 to 0.435	.81	0.018
Experience >10 years	0.007 (0.294)	−0.569 to 0.583	.98	0.004
Perceived diagnostic difficulty: higher	−0.474 (0.089)	−0.648 to −0.301	<.001	−0.257

^a^N=1428 observations from 68 participants and 42 vignette items. Reference categories: correct recommendation, case series A, registered nurse, ≤10 years of experience, and lower perceived difficulty. The case series was included only as a nuisance adjustment. Marginal *R*²=0.176; conditional *R*²=0.500. Standardized effects were obtained from a parallel model in which the outcome and continuous predictors were z-standardized; categorical predictors retained their original coding.

^b^Not applicable.

### Sensitivity Analyses by Case Series

Sensitivity analyses showed that lower trust for incorrect recommendations was evident in both case series: case series A, β=−0.850 (95% CI −1.197 to −0.502), and case series B, β=−0.676 (95% CI −0.996 to −0.356). The longitudinal pattern differed across vignette sets. Trust declined with vignette position in case series A (β=−0.056, 95% CI −0.085 to −0.027) but not in case series B (β=0.004, 95% CI −0.022 to 0.030). In the pooled vignette position × case series model, the case series B slope was 0.064 points per clinical vignette less negative than the case series A slope (95% CI 0.027-0.102). Higher perceived difficulty was negatively associated with trust in both series, whereas intention to use AI was positively associated with trust only in case series B (Table 5).

**Table 5:** Parsimonious models fitted separately within each case series^a^.

Predictor	Series A				Series B		
	β (SE)	95% CI	*P* value	β (SE)		95% CI	*P* value
Intercept	4.918 (0.269)	4.390 to 5.446	<.001	4.846 (0.458)		3.948 to 5.744	<.001
Vignette position (centered)	−0.056 (0.015)	−0.085 to −0.027	<.001	0.004 (0.013)		−0.022 to 0.030	.77
AI recommendation incorrect	−0.850 (0.177)	−1.197 to −0.502	<.001	−0.676 (0.163)		−0.996 to 0.356	<.001
Professional designation: physician	1.144 (0.642)	−0.113 to 2.402	.07	−0.354 (0.564)		−1.459 to 0.752	.53
Baseline trust composite	−0.060 (0.131)	−0.317 to 0.198	.65	0.134 (0.128)		−0.118 to 0.385	.30
Intent to use AI	0.236 (0.203)	−0.163 to 0.634	.25	0.619 (0.211)		0.205 to 1.033	.003
Self-confidence	−0.211 (0.273)	−0.745 to 0.324	.44	0.045 (0.313)		−0.568 to 0.657	.89
Experience >10 years	−0.305 (0.367)	−1.024 to 0.414	.41	0.173 (0.486)		−0.780 to 1.126	.72
Perceived diagnostic difficulty: higher	−0.604 (0.144)	−0.887 to −0.321	<.001	−0.344 (0.103)		−0.546 to −0.141	<.001

^a^Each model included 34 participants, 714 observations, 21 vignette items, and random intercepts for participant and vignette item. Marginal or conditional *R*²=0.178/0.438 for case series A and 0.323/0.601 for case series B. The pooled vignette position × case series model is summarized in the text.

### Exploratory Interaction Model

The exploratory model suggested that sensitivity to incorrect recommendations varied with several characteristics. The incorrect-versus-correct difference was more negative among participants with more than 10 years of experience (interaction β=−0.703, 95% CI −0.998 to −0.407) and at higher self-confidence (interaction β=−0.360 per scale point, 95% CI −0.571 to −0.150), but less negative on clinical vignettes categorized as higher difficulty (interaction β=0.327, 95% CI 0.045-0.609). Interactions with vignette position, baseline trust, and intention to use AI had CIs that included zero. Because this interaction-heavy model was secondary and participant-level power was limited, these findings are considered hypothesis-generating. Full model estimates are reported in Multimedia Appendix 3.

### Exploratory Lagged Model

In the exploratory lagged model, the preceding clinical vignette’s trust composite was positively associated with current-vignette trust (β=0.266, 95% CI 0.210-0.322; standardized effect=0.264). Incorrect current-vignette recommendations remained associated with lower trust (β=−0.753, 95% CI −1.037 to −0.469; standardized effect=−0.405), intention to use AI remained positively associated with trust, and higher perceived difficulty remained negatively associated with trust. Vignette position and the remaining participant-level variables had CIs that included zero. Adding preceding-vignette trust improved model fit relative to the otherwise identical model without the lagged term. Full coefficients and model-fit statistics are reported in Multimedia Appendix 3.

### Hypothesis Summary

H1a was supported because incorrect recommendations received lower vignette-level trust than correct recommendations. H1b was supported statistically because the preceding clinical vignette’s trust composite was positively associated with current-vignette trust, although this result is interpreted as a temporal association rather than evidence of a causal carryover mechanism. H2 was supported because baseline intention to use AI was positively associated with vignette-level trust. H3 was supported because higher perceived diagnostic difficulty was negatively associated with vignette-level trust.

## Discussion

### Principal Findings and Contributions

Participants assigned lower trust to incorrect than to correct AI recommendations, yet accepted half of the incorrect recommendations and rejected approximately 1 in 6 correct recommendations under the stated behavioral coding rule. This aggregate pattern identifies a potential gap between self-reported trust and reliance calibrated to recommendation correctness; it does not establish that individual participants recognized errors before accepting them. Trust in consecutive recommendations was positively associated; baseline intention to use AI was associated with higher trust, and greater perceived difficulty with lower trust. The pooled decline across vignette positions was evident in case series A but not case series B.

Together, these results suggest that clinical AI evaluation should focus on 2 related but distinct questions: whether users can judge the quality of individual AI recommendations and whether those judgments lead to appropriate reliance. The findings should nevertheless be interpreted as preliminary because they were obtained from a small, nurse-dominant sample completing simulated primary care–style tasks.

### Dynamic Trust and Recommendation Correctness

Participants gave substantially lower trust ratings to incorrect recommendations than to correct recommendations. Because the trust questions were completed before current-vignette feedback was shown, this difference suggests that participants were, on average, sensitive to features of the vignette and the apparent plausibility of the AI recommendation. The average difference was therefore present before explicit feedback about that recommendation. This result is consistent with recent repeated-interaction studies. Kahr et al [32] found that people reported greater trust and reliance when AI advice came from a more accurate model, while Yang et al [33] showed that users adjust trust from one interaction to the next and react particularly strongly to automation failures.

The pattern of trust across the 21 clinical vignettes was less consistent. Trust declined in case series A but remained comparatively stable in case series B. This qualification is important because it means that the pooled decline should not be interpreted as a universal process in which trust steadily erodes whenever users encounter AI errors. Instead, the course of trust appears to depend on which cases users see, how convincing the recommendations appear, and where correct and incorrect recommendations occur in the sequence. This result is consistent with Rittenberg et al [34], who found that trust did not always rise and fall in direct proportion to automation reliability. In their experiments, trust sometimes declined even when system reliability improved, and trust was difficult to rebuild after users had experienced a poorly performing system. Kahr et al [32], by contrast, observed increasing trust when participants repeatedly interacted with high-accuracy AI advice. Considered together, these studies and the present findings indicate that there may not be one standard trust trajectory. Trust depends on reliability, but it is also shaped by starting impressions, the order and type of errors, task characteristics, and the user’s ability to evaluate each recommendation. Because recommendation correctness, case content, and vignette position were not independently randomized in this study, the lower trust assigned to incorrect recommendations cannot be attributed solely to correctness. Some of the difference may also reflect the particular cases used or their position in the sequence. The main conclusion is therefore that participants showed aggregate sensitivity to recommendation quality, while the way trust changed over time remained dependent on the specific case series.

### Trust in Consecutive Recommendations

Trust in the immediately preceding clinical vignette was positively associated with trust in the current clinical vignette. In everyday terms, participants appeared to carry part of their recent impression of the AI into the next case rather than evaluating every recommendation in complete isolation. This makes sense in repeated AI use: after seeing several useful recommendations, a person may approach the next recommendation more favorably, whereas recent errors may create greater caution. This finding is consistent with evidence from Kahr et al [32], Yang et al [33], and Rittenberg et al [34] that early reliability experiences can influence later trust and its recovery. This study extends this work to repeated clinical-vignette tasks. However, the lagged finding should not be described as proof of trust inertia. Consecutive ratings from the same participant will often resemble one another potentially because individuals have relatively stable response styles and general attitudes. The most defensible interpretation is that trust showed temporal continuity where the previous rating provided useful information about the next rating, but the study cannot determine whether this occurred because of memory of previous AI performance, accumulated feedback, a stable personal response pattern, or another cognitive process.

The practical implication remains important. Trust in AI should be studied as a developing process rather than as a one-time opinion.

### Reported Trust and Behavioral Reliance

One of the most important contributions of this study is the separate examination of reported trust and actual accept or reject behavior. Lower average trust for incorrect recommendations coexisted with frequent acceptance of those recommendations. This finding supports prior concerns about automation bias in clinical decision support. Gaube et al [35] found that inaccurate advice reduced physicians’ diagnostic accuracy and that substantial subgroups of both radiologists and physicians with less radiology expertise repeatedly followed incorrect advice. This study identifies a related concern in a different task: lower average trust for incorrect advice did not coincide with uniformly low acceptance of that advice. A recommendation may be accepted because it appears plausible, reduces the effort required to reach a decision, or is difficult to verify independently.

At the same time, the present behavioral pattern differs from findings reported by Küper et al [27]. In their 2025 study of 223 dermatologists, participants relied more strongly on correct than incorrect AI advice, but overall reliance on AI was low and self-reliance was high. In this study, by contrast, half of the incorrect recommendations were accepted. This difference may reflect several features: Küper et al [27] studied specialists performing dermatology image-classification tasks, whereas this study included a nurse-dominant sample evaluating short primary care–style vignettes.

The binary accept or reject decision used in this study provides a clear behavioral indicator, but actual clinical reliance may be more complicated. Sivaraman et al [9] found that intensive care unit (ICU) clinicians did not always simply accept or reject AI treatment advice; they often accepted some parts, rejected others, or delayed action, a process described as negotiation. Thus, the current findings clearly demonstrate misalignment between reference correctness and behavior, but future studies should allow more nuanced responses that better reflect how health care professionals use recommendations in practice.

Together, the aggregate findings support measuring trust and behavioral reliance separately rather than assuming that lower self-reported trust ensures rejection of incorrect advice. The present analyses did not directly test a within-participant causal pathway from trust to the accept or reject decision.

### Baseline AI Attitudes and Perceived Difficulty

Participants who entered the study with a stronger intention to use AI generally reported higher trust across the individual cases. This finding is consistent with recent reviews showing that perceived usefulness, openness to technology, previous experience, and expectations about AI influence health care professionals’ acceptance of AI systems [5,6,16]. This study extends that literature by showing that a general willingness to use AI was related not only to overall acceptance, but also to evaluations of specific recommendations during repeated interactions. However, the baseline trust composite itself was not clearly associated with vignette-level trust after the other variables were considered. This suggests that general trust and intention to use AI may not represent the same thing. A participant may express general trust in AI but remain unwilling to use it because of workflow, accountability, or professional concerns. Conversely, someone may be willing to use AI as a helpful tool without assuming that every recommendation is trustworthy. This distinction should be studied more directly in future work.

Participants also reported lower trust in cases they perceived as more difficult. This finding suggests that difficult or ambiguous cases may make participants less certain not only about their own judgment, but also about the quality of the AI recommendation. It complements the finding reported by Gaube et al [35] that the effect of inaccurate clinical advice varied across individual cases and was especially concerning when cases involved difficult-to-recognize diagnostic features. Because perceived difficulty was measured during the same clinical vignette, this study cannot conclude that difficulty caused lower trust. A case may have seemed difficult because the vignette was ambiguous, because the AI recommendation appeared inconsistent, because the participant lacked relevant knowledge, or because several of these factors occurred together. The appropriate takeaway is that participants trusted AI recommendations less on cases they personally experienced as more difficult.

### Limitations and Future Research

Several limitations constrain interpretation. First, the study used short primary care–style clinical vignettes rather than real encounters. The cases were drafted with LLM assistance, anchored to a 2010 textbook, and reviewed by one physician, but were not independently adjudicated by multiple practicing primary care clinicians. They may not reflect current guidelines, contemporary diagnostic pathways, or the complexity of clinical practice.

Second, the sample was small and nurse-dominant. Registered nurses represented 86.76% (59/68) of participants, whereas physicians represented 13.24% (9/68). Although nurses are important users of clinical information and decision support, they do not ordinarily make all primary care diagnoses or prescribe treatments independently. The mismatch between the simulated task and usual RN scope may have affected perceived difficulty, trust, and reliance, limiting ecological validity and generalization to either physician diagnosis or routine nursing practice.

Third, professional designation was self-reported and was not independently verified through licensure, employer, or National Provider Identifier records. The third-party panel did not provide complete counts for invitations, prescreening, or provider-level quality exclusions. Fourth, the participant-level sample limited power for professional-group comparisons and the exploratory interaction tests; their estimates should be interpreted as hypothesis-generating.

Fifth, the two case series differed in vignette content, later correctness positions, and response window. These features were not independently varied, so between-series differences cannot be attributed to response timing or case characteristics. The differing clinical vignette slopes demonstrate that the pooled longitudinal pattern was sensitive to vignette set. Sixth, correctness followed fixed series-specific sequences rather than being randomized or counterbalanced, leaving correctness partly linked to vignette content, vignette position, and reliability phase.

The handling of unrecorded decisions is an additional limitation. Classifying the 50 unrecorded case series B responses as rejection represented nonacceptance, not a verified active decision. It may overstate deliberate rejection and understate acceptance among participants who provided an explicit response. This matters particularly for apparent underreliance: 26 unrecorded responses to correct recommendations contributed to the rejection category. The recorded-response sensitivity summary illustrates this dependence on coding but cannot establish what the unrecorded decisions would have been. Future studies should retain timeout or nonresponse as a separate behavioral outcome rather than assume that it is equivalent to rejection.

### Conclusions

This nurse-dominant pilot study illustrates why clinical AI evaluation should examine both self-reported trust and observed reliance across clinical vignettes. Incorrect recommendations received lower trust on average, but half were accepted; approximately 1 in 6 correct recommendations fell into the rejection category under the stated coding rule. These findings identify a potential evaluation problem rather than a demonstrated patient-safety effect. Larger studies using independently validated, role-aligned clinical vignettes, counterbalanced experimental factors, and separate recording of active rejection and nonresponse are needed to determine how recommendation quality, feedback, and difficulty shape appropriate reliance.

## Appendix

Multimedia Appendix 1 Clinical vignettes, generation procedure, and prompts.

Multimedia Appendix 2 Measurement structure and reliability.

Multimedia Appendix 3 Supplementary Analyses and Results.

## Abbreviations

AVE: average variance extracted
CDSS: clinical decision support systems
CFA: confirmatory factor analysis
CONSORT-AI: Consolidated Standards of Reporting Trials–Artificial Intelligence
ICU: intensive care unit
IRB: Institutional Review Board
LLM: large language model
RN: registered nurse
WVU: West Virginia University
