# Supplementary material for: Clinicians’ Trust in AI-Based Clinical Recommendations Across Controlled Primary Care–Style Clinical Vignettes: Nurse-Dominant Experimental Pilot Study
Source: JMIR Hum Factors. 2026 Oct 8;13:e97649. doi: 10.2196/97649 (PMC13691471; doi:10.2196/97649)
Supplement: Supplementary file 1 [file humanfactors_v13i1e97649_app1.docx]

Multimedia Appendix 1. Clinical Vignettes, Generation Procedure, and Prompts

## Generation and Review Procedure

### Reference Material Before Generation

The authors provided Current Medical Diagnosis & Treatment 2010 [1] to ChatGPT before asking it to generate any study materials. ChatGPT (GPT-4) was used as a drafting tool with the supplied textbook as reference material. This was not a process in which an independently generated diagnosis was accepted solely because the model proposed it: the authors subsequently checked the diagnoses against the book, and an expert physician performed the final review.

### ChatGPT-Generated Clinical Vignettes and Initial Diagnoses

ChatGPT generated short fictional clinical vignettes and associated initial diagnoses and recommendations. The materials represented common adult presentations within a primary care-style scope and were intended to be understandable without subspecialty knowledge. Vignette information included the presenting symptoms and relevant clinical context needed to evaluate a recommendation. The material was not taken from real patient records. The final patient descriptions and participant-facing recommendations, rather than newly generated substitutes, are reproduced in Tables S1 and S2.

### Author Review Against the Textbook

The authors reviewed the generated materials first. They compared the proposed diagnoses with the textbook and checked whether the patient information and intended diagnosis were clinically coherent. They also examined readability, completeness, internal consistency, obvious contradictions, and the relationship between the recommendation shown to the participant and its intended correctness status. No corrections to the initial reference diagnoses were required at this stage.

### Expert Physician Review and Finalization

After the authors’ comparison with the textbook, a physician reviewed the clinical vignettes, diagnoses, and recommendations before deployment. Her review focused on clinical plausibility, whether the supplied clinical information supported the intended reference diagnosis, and whether a participant-facing recommendation appropriately represented its designated correct or intentionally incorrect status. She finalized the reference diagnoses and approved the materials for the experiment. No corrections to the initial reference diagnoses were required during this physician review.

### Reference Diagnoses and Intentionally Incorrect Recommendations

A reference diagnosis and a participant-facing AI recommendation served different functions. The reference diagnosis defined the study’s intended diagnostic answer. The participant-facing recommendation was either consistent with that answer or deliberately incorrect, depending on the programmed experimental condition for the vignette. Intentionally incorrect alternatives were retained as experimental stimuli and should not be interpreted as correct clinical guidance or as evidence that inaccurate initial reference diagnoses passed review. The physician reviewed the materials in light of this distinction.

### Assembly and Pilot Testing

The final materials comprised two series of 21 clinical vignettes, each with 10 correct and 11 intentionally incorrect recommendations. The series-specific order and correctness labels are reproduced in Tables S1 and S2. The first 20 eligible participants formed the participant pilot test; they followed the same procedures as the remainder of the final sample. No technical or comprehension problems were identified and no experimental changes were made after the pilot test.

## Reference

1. McPhee SJ, Papadakis MA, editors. Current Medical Diagnosis & Treatment 2010. 49th ed. New York, NY: McGraw-Hill Medical; 2010.

## Prompt Templates

### Prompt Template 1:

I have provided Current Medical Diagnosis & Treatment 2010 as reference material for a research task. Use this book as the medical reference when I ask you to generate fictional clinical vignettes and associated diagnoses and recommendations. The materials are for a controlled study of how healthcare professionals evaluate AI recommendations, not for the care of real patients. Do not generate any vignette yet. Do not claim that an answer is supported by the supplied book if you cannot establish that support.

### Prompt Template 2:

Using the medical reference already provided, draft fictional primary care-style clinical vignettes involving common adult presentations. The study materials will form two series of 21 clinical vignettes. Keep the scope and expected difficulty broadly comparable and avoid presentations that require subspecialty knowledge. For each vignette, provide a fictional patient description, the presenting concern, relevant symptoms and history, and other clinically relevant information needed to evaluate a diagnostic recommendation. Include vital signs, medication history, or social context only when appropriate to the presentation. Keep the information readable, complete enough for the research task, and internally consistent. Do not use identifiable real-patient information. Keep each vignette identifiable so that its associated diagnosis and recommendation can be reviewed separately.

### Prompt Template 3:

For each fictional clinical vignette, use the supplied medical reference to propose the most appropriate reference diagnosis and a concise clinical recommendation. Keep the patient information unchanged while evaluating the diagnosis. Return the vignette identifier, the proposed reference diagnosis, a brief explanation of how the presentation supports that diagnosis, and a separately labeled participant-facing diagnosis and recommendation. If the clinical information or supplied reference is insufficient to support a diagnosis, flag that uncertainty for human review rather than filling the gap with an unsupported conclusion. These are drafts: the study authors will compare the diagnoses with the book, and an expert physician will review and finalize the materials.

### Prompt Template 4:

For the clinical vignettes designated to receive an intentionally incorrect AI recommendation, retain the vignette and its reference diagnosis and draft an alternative diagnosis and associated recommendation that are deliberately inconsistent with the intended reference answer. This alternative is an experimental stimulus, not clinical advice. Keep the reference diagnosis separate from the diagnosis and recommendation that will be shown to participants. For vignettes designated correct, retain a participant-facing recommendation consistent with the reference diagnosis. Return the vignette identifier, reference diagnosis, participant-facing recommendation, and intended correctness status as separately labeled fields for author and physician review. Do not reveal the correctness label within the participant-facing recommendation; correctness feedback will be presented separately after participants submit their responses.

## Interpretation and Reproducibility

The reference book was supplied before generation, the authors performed the first diagnostic comparison, and the expert physician performed the final clinical review. No initial reference diagnosis required correction during those reviews. This review outcome does not establish perfect clinical accuracy, agreement among independent physicians, or consistency with all contemporary guidelines. The study used one physician reviewer and did not calculate inter-rater agreement.

Due to nature of generative AI and large language models, repeating the prompts is not claimed to reproduce the administered text verbatim. The final materials in Tables S1 and S2 are the record of what was presented to participants.

## Clinical Vignettes Presented to Participants

All patient names and clinical scenarios below are fictional. Intentionally incorrect recommendations were used only as experimental stimuli and are not clinical guidance. The patient descriptions and AI recommendations are reproduced as presented to study participants; correctness labels identify their programmed experimental status.

## Table S1. Case Series A (no fixed response window)

| **Trial** | **Patient vignette** | **AI diagnosis and recommendation** | **Programmed status** |
| --- | --- | --- | --- |
| 1 | Maria Garcia, a 43-year-old woman, reports persistent headaches, dizziness, and fatigue, particularly in the evenings. Her initial vitals indicate high blood pressure (160/100 mmHg). She has a family history of hypertension and diabetes and is currently taking lisinopril for hypertension management, along with occasional ibuprofen for headaches. Maria lives in a low-income rural area in Texas, works as a housekeeper, and has limited access to healthcare due to a lack of insurance. | Diagnosis: Uncontrolled Systemic Hypertension. Recommendation: Maria Garcia presents with uncontrolled Stage 2 hypertension (BP 160/100 mmHg) and associated symptoms of headache, dizziness, and fatigue. Management includes titrating lisinopril or adding a complementary antihypertensive agent, implementing lifestyle modifications (weight management, DASH diet, sodium restriction, physical activity), and monitoring for hypertensive complications. Patient education regarding adherence and warning signs is crucial. | Correct |
| 2 | James Peterson, a 25-year-old male, presents with a mild sore throat, low-grade fever, and a dry, persistent cough lasting for three days. His initial vitals are within normal limits, with a blood pressure of 118/76 mmHg, heart rate of 82 bpm, and temperature of 98.4°F. He reports no chronic medical conditions and is generally in good health. Occasionally, he uses throat lozenges for irritation. James leads a sedentary lifestyle as a full-time software developer but engages in occasional weekend hikes. He has employer-provided health insurance and visits a nearby community clinic for healthcare needs. | Diagnosis: Viral Upper Respiratory Tract Infection (URI) Recommendation: James Peterson’s presentation of mild sore throat, low-grade fever, and dry persistent cough is consistent with a viral upper respiratory tract infection. Management should focus on symptomatic care including the use of throat lozenges, warm saline gargles, hydration, rest, and over-the-counter analgesics like acetaminophen or ibuprofen. Antibiotic therapy is not indicated unless symptoms worsen or new signs of bacterial infection develop. | Correct |
| 3 | Sarah Bennett, a 29-year-old female, presents with persistent itching and red, scaly patches on her arms and hands that have lasted for two weeks. Her initial vitals are within normal ranges, including a blood pressure of 120/78 mmHg, heart rate of 75 bpm, and temperature of 98.6°F. She has a family history of eczema and was diagnosed with mild eczema in childhood. Sarah occasionally uses over-the-counter hydrocortisone cream for symptom relief. Given her occupation as a teacher, frequent exposure to chalk and cleaning supplies may be exacerbating her symptoms. She has regular access to healthcare through her employer-provided insurance and visits a primary care clinic when needed. | Diagnosis: Atopic Dermatitis (Eczema) exacerbation Recommendation: Sarah Bennett’s presentation of persistent itching and red, scaly patches on her arms and hands is consistent with an exacerbation of atopic dermatitis (eczema). Management should include mid- to high-potency topical corticosteroids, liberal use of moisturizers, avoidance of known irritants like cleaning supplies, and patient education on gentle skin care practices. If symptoms persist despite appropriate topical therapy, escalation to non-steroidal immunomodulators or dermatology referral may be considered. | Correct |
| 4 | James Taylor, a 35-year-old male, presents with persistent abdominal discomfort characterized by bloating, alternating constipation and diarrhea lasting for two weeks. His initial vitals are within normal limits, with a blood pressure of 125/82 mmHg, heart rate of 80 bpm, and temperature of 98.7°F. He has a family history of irritable bowel syndrome (IBS) and reports occasional episodes of indigestion. His lifestyle includes a diet high in processed foods and limited physical activity. He occasionally uses antacids for indigestion and has access to a local primary care clinic through his employer-provided health insurance. | Diagnosis: Irritable bowel syndrome (IBS) Recommendation: James Taylor’s symptoms of persistent bloating, alternating constipation and diarrhea are consistent with irritable bowel syndrome (IBS). Management should focus on patient education and reassurance, identification and avoidance of dietary triggers (e.g., fatty foods, caffeine, sorbitol, fructose), symptomatic treatment with antispasmodics, loperamide for diarrhea, and polyethylene glycol for constipation. Regular exercise and stress management techniques should also be encouraged. Ongoing monitoring is necessary to detect any emerging alarm symptoms. | Correct |
| 5 | Sarah Mitchell is a 34-year-old non-smoking female residing in a suburban home in San Diego, California. She reports a sore throat, mild fever, swollen lymph nodes in the neck, and difficulty swallowing that has persisted for three days. Her initial vitals are within normal range: blood pressure 110/70 mmHg, heart rate 65 bpm, respiratory rate 14 bpm, and temperature 98.1°F. Sarah occasionally takes acetaminophen for mild pain relief and has no significant medical history aside from seasonal allergies. She works as a kindergarten teacher, which increases her exposure to respiratory infections. | Diagnosis: Retinal detachment. Recommendation: Prompt referral to an ophthalmologist is essential, as early surgical intervention can significantly reduce the risk of permanent vision loss. Delays in treatment may lead to irreversible damage, making urgent evaluation and management critical. | Incorrect |
| 6 | John Peterson, a 32-year-old male, presents with persistent bloating, abdominal cramps, and loose stools after consuming meals containing dairy products. His initial vitals are within normal limits, with a blood pressure of 124/82 mmHg and a temperature of 98.6°F. He has a family history of lactose intolerance and no major past illnesses, though he occasionally experiences mild heartburn. His lifestyle as a software engineer involves irregular meal timings and frequent consumption of fast food. To manage his symptoms, he occasionally takes antacids for heartburn relief. | Diagnosis: Irritable Bowel Syndrome (IBS). Recommendation: John Peterson’s symptoms of abdominal cramps, bloating, and alternating bowel habits are consistent with irritable bowel syndrome (IBS). Initial management should include dietary changes such as increasing fiber intake, along with behavioral modifications and reassurance. Antispasmodic medications (e.g., dicyclomine) may be used for symptom relief, especially in cases of abdominal pain or cramping. A food diary can help identify dietary triggers. | Incorrect |
| 7 | Sarah Thompson, a 45-year-old female, reports morning stiffness, swelling, and joint pain in her hands and knees, which interfere with her daily activities. Her initial vitals are within the normal range, and she has a family history of rheumatoid arthritis. She has no major past illnesses but occasionally experiences mild joint pain after physical activity. She takes acetaminophen occasionally for joint pain relief. Given her symptoms and medical history, further evaluation for rheumatoid arthritis is warranted. | Diagnosis: Rheumatoid Arthritis (RA). Recommendation: Sarah Thompson’s symptoms of morning stiffness, swelling, and joint pain in the hands and knees are highly suggestive of rheumatoid arthritis. Diagnostic workup should include rheumatoid factor, anti-CCP antibodies, ESR, and CRP levels, along with radiographs of the hands and feet. Early initiation of DMARD therapy such as methotrexate and NSAIDs for symptom control are recommended. Early referral to a rheumatologist is essential for confirmation and treatment optimization. | Correct |
| 8 | Michael Reed, a 32-year-old male, reports persistent sadness, fatigue, and lack of motivation lasting over two months, along with insomnia. His initial vitals are within normal limits: blood pressure of 120/80 mmHg, heart rate of 68 bpm, respiratory rate of 14 bpm, and temperature of 98.2°F. He has a family history of depression and was previously diagnosed with mild anxiety. His sedentary lifestyle and long working hours as a financial analyst may contribute to his symptoms. He occasionally takes melatonin for sleep but has no current prescription medications. Given the chronic nature of his symptoms and difficulty maintaining daily functioning, appropriate intervention is necessary. | Diagnosis: Insomnia. Recommendation: Michael Reed’s ongoing insomnia, fatigue, and low mood may indicate a sleep disturbance contributing to his depressive symptoms. Short-term use of a sleep aid like zolpidem can help restore a regular sleep pattern, while relaxation techniques such as deep breathing or guided meditation may support long-term improvement. A follow-up in six weeks is important to assess effectiveness and monitor for dependence or residual symptoms. | Incorrect |
| 9 | Olivia Carter, a 29-year-old female, reports recurring headaches, intermittent blurry vision, and a sensation of pressure in her head, particularly during work hours. She experiences occasional nausea and difficulty focusing. Her initial vitals are within normal ranges, with a blood pressure of 115/75 mmHg and a heart rate of 72 bpm. She has no history of migraines but has a family history of hypertension. Olivia works as a graphic designer and spends long hours in front of a computer screen, occasionally engaging in light exercise. She has a history of mild anxiety and occasionally takes ibuprofen for headache relief. | Diagnosis: Tension Headaches Recommendation: Olivia Carter’s symptoms of recurring headaches, intermittent blurry vision, and a sensation of pressure—especially during prolonged screen use—are most consistent with tension-type headache, likely aggravated by visual strain and poor posture. Management includes simple analgesics such as ibuprofen, ergonomic corrections, screen breaks, and light physical activity. Further evaluation by an ophthalmologist may be warranted if visual disturbances persist or worsen. | Correct |
| 10 | Emma Wilson is a 36-year-old woman living in a shared apartment in Seattle, Washington. She works as a full-time barista, often standing for long hours during her shifts. She presents with complaints of persistent fatigue, cold intolerance, and unintentional weight gain over the past three months. Her initial vitals show a blood pressure of 110/70 mmHg, heart rate of 60 bpm, respiratory rate of 14 bpm, and temperature of 97.6°F. She has a history of mild depression and seasonal allergies but no chronic illnesses. Emma reports irregular menstrual cycles and dry skin. She occasionally takes loratadine for allergies and does not smoke or drink alcohol. She has moderate access to healthcare through her employer-provided insurance and reports generally good hygiene practices. Emma is in a long-term monogamous relationship, identifies as heterosexual, and has no significant family history of endocrine disorders. | Diagnosis: Iron-deficiency anemia. Recommendation: Based on Emma Wilson's conditions indicating Iron-deficiency anemia, prescribe oral iron supplements and recommend increasing dietary intake of iron-rich foods such as red meat, spinach, and legumes. Suggest follow-up in 8 weeks to reassess blood counts. | Incorrect |
| 11 | Ethan Rodriguez, a 34-year-old male, reports mild to moderate ear pain, occasional muffled hearing, and a sensation of fullness in his right ear for the past three days. His initial vitals are stable, with a blood pressure of 120/80 mmHg, heart rate of 78 bpm, respiratory rate of 16 bpm, and temperature of 98.7°F. He has a history of mild seasonal allergies and takes antihistamines occasionally. Ethan works as an IT specialist, spending long hours at a desk, and enjoys outdoor activities such as hiking and cycling on weekends. His healthcare accessibility is good through private insurance. | Diagnosis: Eustachian Tube Dysfunction (ETD), possibly progressing to Serous Otitis Media (SOM) Recommendation: Ethan Rodriguez’s symptoms of mild ear pain, fullness, and intermittent muffled hearing are consistent with eustachian tube dysfunction (ETD), likely secondary to allergic inflammation. Management includes autoinflation, oral or topical decongestants, and intranasal corticosteroids for allergy control. Reassessment in 1–2 weeks is advised, and ENT referral may be needed if symptoms persist. | Correct |
| 12 | Olivia Martinez, a 52-year-old female, reports experiencing episodes of chest discomfort and shortness of breath during mild physical activities over the past week. Her initial vitals show a blood pressure of 138/85 mmHg, a heart rate of 98 bpm, and a respiratory rate of 18 bpm. She has a family history of cardiovascular disease and hypertension and was previously diagnosed with pre-hypertension. Olivia is a former smoker who quit 10 years ago and occasionally takes aspirin for mild headaches. She works as a school teacher, engages in moderate physical activity, and follows a relatively balanced diet. Given her symptoms and medical background, further evaluation and intervention are necessary to assess and manage her condition effectively | Diagnosis: Gastroesophageal Reflux Disease (GERD) Recommendation: For Olivia Martinez’s symptoms, it is recommended to prescribe a daily proton pump inhibitor (PPI), such as omeprazole, to reduce gastric acid production and relieve chest discomfort. In addition, advise Olivia to make dietary modifications, including avoiding spicy, fatty, and acidic foods. A follow-up appointment should be scheduled in 1 month to assess symptom improvement and adjust treatment if necessary. | Incorrect |
| 13 | David Martinez, a 52-year-old insured male with moderate healthcare access, presents with yellowing of the skin and eyes (jaundice), upper right abdominal discomfort, fatigue, and occasional nausea. His initial vitals are within normal limits, and he has a history of hypertension and previously elevated liver enzymes. He occasionally takes acetaminophen for headaches and consumes alcohol socially. The patient reports a family history of liver disease and currently takes lisinopril for blood pressure management. | Diagnosis: Gallbladder disease. Recommendation: For David Martinez’s presentation, it is recommended to proceed with a cholecystectomy based on a presumed diagnosis of gallstones. An immediate surgical consultation should be arranged, along with preoperative imaging such as an abdominal ultrasound to confirm gallstone presence before surgery. David should be advised to fast and prepare for potential surgical intervention within the next 24 to 48 hours to prevent possible complications. | Incorrect |
| 14 | Emily Dawson is a 31-year-old female living in downtown Denver. She works as a barista and maintains a healthy BMI of 23.4. Her vital signs are within normal range, and she has access to healthcare through employer-provided insurance. For the past three days, she has experienced a burning sensation during urination, increased frequency, and mild pelvic pain. Her primary complaint is painful and frequent urination. She occasionally takes ibuprofen for menstrual cramps but has no chronic illnesses or surgical history. Her sexual health history is unremarkable, and she maintains good hygiene practices. She has no physical or mental limitations and lives with her pet cat. | Diagnosis: Menstrual cramps Recommendation: : For Emily Dawson’s symptoms, it is recommended to manage presumed menstrual cramps by advising rest and encouraging the continued use of over-the-counter ibuprofen as needed for pain relief. Additionally, applying warm compresses to the lower abdomen is suggested to alleviate discomfort. Emily should be advised to monitor her symptoms over the next few days, with the expectation that they will resolve spontaneously without further intervention. | Incorrect |
| 15 | Daniel Foster, a 42-year-old male, reports persistent knee pain and stiffness, particularly after periods of inactivity or physical activity. His initial vitals are within normal ranges, and he has a family history of osteoarthritis. Diagnosed with mild osteoarthritis in his knees two years ago, he occasionally uses ibuprofen for pain relief. His lifestyle includes a sedentary IT job but active participation in recreational soccer on weekends. Given his symptoms and medical history, appropriate management focuses on improving joint mobility and reducing discomfort. | Diagnosis: Rheumatoid arthritis Recommendation: For Daniel Foster’s symptoms, it is recommended to initiate treatment for rheumatoid arthritis by prescribing methotrexate to control underlying inflammation and prevent joint damage. Concurrently, a low-dose corticosteroid should be prescribed to provide more immediate relief of symptoms. Regular blood tests are advised to monitor potential side effects of methotrexate, including liver function and blood cell counts. | Incorrect |
| 16 | Rachel Simmons, a 35-year-old woman, reports persistent lower back pain, especially in the lumbar region, which worsens with prolonged sitting or heavy lifting. She has a history of mild degenerative disc disease diagnosed a year ago, with previous physical therapy yielding limited improvement. Her vitals are within normal limits, and she occasionally takes acetaminophen for pain relief. She works as an accountant, leading a mostly sedentary lifestyle, though she engages in yoga twice a week. Her family history includes degenerative disc disease in her father. | Diagnosis: Rheumatoid arthritis. Recommendation: Based on Rachel Simmons’ reported lower back pain and her family history of degenerative disc disease, the AI has diagnosed her with rheumatoid arthritis. It is recommended to initiate treatment with methotrexate to address presumed autoimmune inflammation and prevent joint deterioration. In addition, a corticosteroid regimen should be prescribed to provide symptom relief during the early phase of treatment. Regular blood tests should be conducted to monitor for potential side effects, such as liver toxicity or immune suppression. A follow-up appointment is advised within 2 weeks to reassess treatment response and modify dosage as needed. | Incorrect |
| 17 | Daniel Thompson, a 27-year-old male with no significant medical history and excellent access to healthcare, reports intense throat pain, difficulty swallowing, and mild fever for the past three days. His initial vitals include a temperature of 100.4°F and stable blood pressure and heart rate. Symptoms include white patches on the tonsils and swollen, tender lymph nodes in the neck. He occasionally experiences seasonal allergies and took acetaminophen to manage the fever. Daniel lives an active lifestyle, works in software development, and has no physical or mental limitations. | Diagnosis: Streptococcal pharyngitis (strep throat) Recommendation: Daniel Thompson’s symptoms of intense throat pain, difficulty swallowing, swollen lymph nodes, and tonsillar exudates are consistent with group A β-hemolytic streptococcal pharyngitis. Management includes a rapid antigen test or throat culture confirmation, followed by penicillin VK or amoxicillin for 10 days if positive. Symptomatic relief with acetaminophen and saline gargles is advised. Prompt treatment is essential to prevent complications such as rheumatic fever. | Correct |
| 18 | Michael Johnson, a 48-year-old male, reports persistent lower back pain and frequent urination, particularly at night. His initial vitals indicate elevated blood pressure (145/95 mmHg) and a heart rate of 85 bpm. He has a family history of hypertension and kidney disease and has been managing hypertension with lisinopril. His sedentary lifestyle and high-sodium diet may contribute to his symptoms. He occasionally takes ibuprofen for back pain. Given his symptoms and medical background, further evaluation is required to assess potential kidney function issues. | Diagnosis: Chronic Kidney Disease (CKD). Recommendation: Michael Johnson’s persistent lower back pain, nocturia, hypertension, and family history suggest early chronic kidney disease, possibly secondary to hypertensive nephrosclerosis. Management includes ordering serum creatinine, BUN, urinalysis, and renal ultrasound, optimizing blood pressure control, discontinuing NSAIDs, encouraging a low-sodium diet, and arranging nephrology referral if kidney function worsens. | Correct |
| 19 | Kevin Brooks is a 36-year-old male living in Atlanta, Georgia, with a BMI of 27.1. He reports sharp pain in the lower right abdomen, nausea, and a low-grade fever. His initial vitals include a blood pressure of 120/80 mmHg, heart rate of 76 bpm, and a temperature of 98.6°F. His primary complaint is severe localized abdominal pain. He has no chronic illnesses or prior surgeries and occasionally takes ibuprofen. Kevin has moderate access to healthcare through employer-provided insurance, works as a retail manager, and reports no physical or mental limitations. | Diagnosis: Gastroenteritis Recommendation: Based on Kevin Brooks’ symptoms of sharp lower right abdominal pain, nausea, and a low-grade fever, he is diagnosed with gastroenteritis. It is recommended that Kevin rest at home, maintain hydration with electrolyte-rich fluids, and take over-the-counter antidiarrheal medications to manage gastrointestinal symptoms. He should avoid heavy meals and monitor for any worsening of symptoms. A follow-up consultation is advised in one week if symptoms do not improve. | Incorrect |
| 20 | Laura Thompson, a 35-year-old female, presents with a persistent dry cough lasting for three weeks, accompanied by mild shortness of breath and fatigue, particularly after physical exertion. Her initial vitals are within normal ranges, with a temperature of 99°F. She has a family history of asthma but no personal history of chronic respiratory conditions. She has previously been diagnosed with seasonal allergies and occasionally takes antihistamines. Laura works as a graphic designer, leading a mostly sedentary lifestyle with occasional yoga sessions. | Diagnosis: Cough-Variant Asthma Recommendation: Laura Thompson’s three-week dry cough with mild exertional dyspnea and family history of asthma is consistent with cough-variant asthma. Initial management includes a 2–4 week trial of inhaled β2-agonist therapy, followed by spirometry or methacholine challenge if symptoms persist. Antihistamines or nasal corticosteroids may be used if postnasal drip is suspected. | Correct |
| 21 | Amanda Lewis is a 26-year-old female graduate student living in Seattle with moderate access to healthcare through student insurance. She presents with painful urination, frequent bathroom visits, and lower abdominal discomfort. Her initial vitals are normal (BP: 112/72 mmHg, HR: 78 bpm, Temp: 98.9°F). She has no chronic conditions or surgical history, but her mother has a history of recurrent UTIs. Amanda occasionally takes ibuprofen and maintains good sexual hygiene. She is sexually active with one partner, a non-smoker, and reports no physical or mental limitations. | Diagnosis: Vaginal yeast infection Recommendation: Based on Amanda Lewis’s reported symptoms of painful urination, frequent bathroom visits, and lower abdominal discomfort, the AI has diagnosed her with a vaginal yeast infection. It recommends initiating antifungal treatment such as fluconazole and advises Amanda to avoid wearing tight-fitting clothing and synthetic underwear to reduce moisture and irritation. A follow-up appointment in one week is suggested to reassess symptom resolution and rule out persistent infection. | Incorrect |
