# Supplementary material for: Clinicians’ Trust in AI-Based Clinical Recommendations Across Controlled Primary Care–Style Clinical Vignettes: Nurse-Dominant Experimental Pilot Study
Source: JMIR Hum Factors. 2026 Oct 8;13:e97649. doi: 10.2196/97649 (PMC13691471; doi:10.2196/97649)
Supplement: Supplementary file 2 [file humanfactors_v13i1e97649_app2.docx]

Multimedia Appendix 2. Measurement Structure and Reliability

Vignette-level trust was measured at each clinical vignette with three ordinal indicators: perceived trust in the AI recommendation, perceived transparency of the recommendation, and likelihood of acting on the recommendation. Vignette-specific one-factor confirmatory summaries were aligned to the actual 21 clinical-vignette positions. Because each three-indicator model was just-identified (df = 0), global fit indices were not used to judge model fit. Evidence was summarized using standardized loading estimates from polychoric correlation matrices, average variance extracted (AVE), coefficient omega, and Cronbach’s alpha.

Table S1. Summary of measurement evidence.

| **Measure** | **Indicators/data** | **AVE** | **Coefficient ω** | **Cronbach’s α** |
| --- | --- | --- | --- | --- |
| Self-confidence | 9 retained baseline items | .65 | .94 | .91 |
| Baseline trust composite | 3 baseline indicators; reliability based on 66 complete cases | — | — | .938 |
| Vignette-level trust composite | 3 indicators at each of 21 actual clinical vignettes | .761–.922 | .904–.973 | .868–.962 |

Note. Two participants lacked the baseline transparency item; their baseline trust composites were calculated from the two available indicators. The baseline alpha is reported for the 66 participants with all three indicators.

Table S2. Vignette-specific trust measurement estimates.

| **Vignette** | **Trust loading** | **Transparency loading** | **Likelihood-to-act loading** | **AVE** | **ω** | **α** |
| --- | --- | --- | --- | --- | --- | --- |
| 1 | 0.930 | 0.746 | 0.927 | 0.761 | 0.904 | 0.868 |
| 2 | 0.992 | 0.822 | 0.910 | 0.830 | 0.936 | 0.907 |
| 3 | 0.962 | 0.793 | 0.911 | 0.795 | 0.920 | 0.896 |
| 4 | 0.939 | 0.920 | 0.987 | 0.901 | 0.965 | 0.945 |
| 5 | 0.986 | 0.791 | 0.968 | 0.845 | 0.942 | 0.901 |
| 6 | 0.983 | 0.879 | 0.936 | 0.872 | 0.953 | 0.938 |
| 7 | 0.970 | 0.904 | 0.959 | 0.893 | 0.962 | 0.947 |
| 8 | 0.870 | 0.851 | 1.034 | 0.850 | 0.944 | 0.918 |
| 9 | 0.963 | 0.842 | 0.983 | 0.868 | 0.951 | 0.934 |
| 10 | 0.974 | 0.780 | 0.973 | 0.834 | 0.937 | 0.909 |
| 11 | 0.991 | 0.826 | 0.912 | 0.832 | 0.937 | 0.911 |
| 12 | 0.984 | 0.867 | 0.970 | 0.887 | 0.959 | 0.941 |
| 13 | 0.925 | 0.895 | 0.974 | 0.868 | 0.952 | 0.928 |
| 14 | 0.982 | 0.823 | 0.994 | 0.876 | 0.955 | 0.928 |
| 15 | 0.953 | 0.697 | 0.981 | 0.785 | 0.915 | 0.869 |
| 16 | 0.966 | 0.902 | 0.985 | 0.905 | 0.966 | 0.946 |
| 17 | 1.012 | 0.900 | 0.965 | 0.922 | 0.973 | 0.962 |
| 18 | 0.981 | 0.894 | 0.955 | 0.891 | 0.961 | 0.947 |
| 19 | 0.995 | 0.804 | 0.948 | 0.845 | 0.942 | 0.916 |
| 20 | 0.967 | 0.869 | 0.950 | 0.865 | 0.950 | 0.930 |
| 21 | 0.981 | 0.760 | 0.889 | 0.777 | 0.912 | 0.882 |

Note. Standardized loadings were estimated from vignette-specific polychoric correlation matrices. The three-indicator models were just-identified, so chi-square and global fit indices are not informative. At Clinical Vignettes 8 and 17, one loading estimate was slightly above 1.0 (1.034 and 1.012, respectively), a small-sample Heywood pattern; those estimates are reported transparently and the corresponding reliability coefficients remained high.

## Self-Confidence Measurement

The nine retained self-confidence items formed a one-factor measure with AVE = .65, coefficient ω = .94, and Cronbach’s α = .91. These indices support convergent measurement and internal consistency for the retained self-confidence score.
