# Supplementary material for: Clinicians’ Trust in AI-Based Clinical Recommendations Across Controlled Primary Care–Style Clinical Vignettes: Nurse-Dominant Experimental Pilot Study
Source: JMIR Hum Factors. 2026 Oct 8;13:e97649. doi: 10.2196/97649 (PMC13691471; doi:10.2196/97649)
Supplement: Supplementary file 3 [file humanfactors_v13i1e97649_app3.docx]

Multimedia Appendix 3. Supplementary Results

This appendix provides clinical-vignette distributions, exploratory interaction and lagged-model estimates, a descriptive sensitivity analysis of unrecorded accept/reject responses, and an audit of their associated page-submit timing records.

## Descriptive Distributions

Figure S1. Number of participants rating each clinical vignette as lower difficulty (Very easy/Easy) or higher difficulty (Moderate/Difficult/Very difficult). Asterisks identify intentionally incorrect recommendations according to the series-specific programmed sequence.

Figure S2. Mean vignette-level trust composites by clinical-vignette position. Error bars indicate 95% confidence intervals. Asterisks identify intentionally incorrect recommendations.

Figure S3. Mean vignette-level trust composite by case series, perceived diagnostic difficulty, and AI recommendation correctness. Error bars indicate 95% confidence intervals.

Figure S4. Acceptance and rejection-category counts by clinical-vignette position. Asterisks identify intentionally incorrect recommendations. The Case Series B rejection category includes 50 unrecorded responses; this category is not limited to explicit rejection.

## Exploratory Models

Table S1. Exploratory cross-classified model including AI-incorrectness interactions for the vignette-level trust composite.

| **Predictor** | **β** | **SE** | **95% CI** | **p** |
| --- | --- | --- | --- | --- |
| Intercept | 4.641 | 0.259 | 4.134 to 5.149 | <.001 |
| Case Series B vs A (nuisance adjustment) | 0.181 | 0.304 | −0.415 to 0.777 | .551 |
| Professional designation: physician | 0.241 | 0.403 | −0.548 to 1.030 | .550 |
| AI recommendation incorrect | −0.547 | 0.181 | −0.902 to −0.191 | .003 |
| Experience >10 years | 0.378 | 0.306 | −0.221 to 0.977 | .216 |
| Perceived diagnostic difficulty: higher | −0.644 | 0.115 | −0.869 to −0.419 | <.001 |
| Incorrect AI × experience >10 years | −0.703 | 0.151 | −0.998 to −0.407 | <.001 |
| Incorrect AI × higher perceived difficulty | 0.327 | 0.144 | 0.045 to 0.609 | .023 |
| Vignette position (centered) | −0.038 | 0.015 | −0.067 to −0.010 | .009 |
| Incorrect AI × vignette position | 0.031 | 0.024 | −0.016 to 0.078 | .198 |
| Baseline trust composite | 0.024 | 0.099 | −0.170 to 0.219 | .806 |
| Incorrect AI × baseline trust | 0.090 | 0.052 | −0.011 to 0.191 | .081 |
| Intent to use AI | 0.316 | 0.150 | 0.023 to 0.609 | .035 |
| Incorrect AI × intent to use AI | 0.145 | 0.078 | −0.007 to 0.297 | .062 |
| Self-confidence | 0.232 | 0.207 | −0.173 to 0.637 | .261 |
| Incorrect AI × self-confidence | −0.360 | 0.107 | −0.571 to −0.150 | <.001 |

Note. N = 1,428 observations. Random intercepts were included for participant and vignette item. Case series was included only as a nuisance adjustment. Marginal R² = .194; conditional R² = .524. Interaction coefficients are exploratory and should not be interpreted as confirmatory subgroup effects.

Table S2. Exploratory lagged cross-classified model predicting the current-vignette trust composite.

| **Predictor** | **β** | **SE** | **95% CI** | **p** | **Std. effect** |
| --- | --- | --- | --- | --- | --- |
| Intercept | 4.810 | 0.207 | 4.403 to 5.217 | <.001 | — |
| Preceding-vignette trust composite (centered) | 0.266 | 0.028 | 0.210 to 0.322 | <.001 | 0.264 |
| Vignette position (centered) | −0.016 | 0.013 | −0.041 to 0.008 | .191 | −0.051 |
| AI recommendation incorrect | −0.753 | 0.145 | −1.037 to −0.469 | <.001 | −0.405 |
| Case Series B vs A (nuisance adjustment) | 0.172 | 0.242 | −0.303 to 0.647 | .479 | 0.092 |
| Professional designation: physician | 0.195 | 0.300 | −0.393 to 0.783 | .516 | 0.105 |
| Baseline trust composite | 0.042 | 0.071 | −0.097 to 0.182 | .552 | 0.037 |
| Intent to use AI | 0.286 | 0.108 | 0.074 to 0.497 | .008 | 0.176 |
| Self-confidence | 0.015 | 0.148 | −0.275 to 0.305 | .920 | 0.006 |
| Experience >10 years | −0.018 | 0.220 | −0.449 to 0.413 | .935 | −0.010 |
| Perceived diagnostic difficulty: higher | −0.486 | 0.089 | −0.660 to −0.311 | <.001 | −0.261 |

Note. The model included 1,360 observations from Clinical vignettes 2–21. Random intercepts were included for participant and vignette item. Marginal R² = .260; conditional R² = .470. The maximum-likelihood comparison favored the model containing preceding-vignette trust, likelihood-ratio χ²(1) = 92.93, p < .001; Akaike information criterion (AIC) decreased from 4867.22 to 4776.29.

## Recorded-Response-Only Sensitivity Analysis

The primary descriptive analysis places unrecorded Case Series B decisions in the rejection category, representing absence of recorded acceptance rather than verified active rejection. The recorded-response summary instead excludes those observations. Neither approach reveals what the missing decisions would have been; differences reflect both classification and denominator changes. This is a descriptive sensitivity comparison, not a missing-at-random correction or a new trust-model analysis.

Table S3. Behavioral summaries under rejection-category coding and recorded-response-only reporting.

| **Case series and coding** | Included observations, n | **Accepted incorrect, n/N (%)** | **Rejected correct, n/N (%)** |
| --- | --- | --- | --- |
| A: Rejection-category coding | 714 | 185/374 (49.47%) | 52/340 (15.29%) |
| A: Recorded responses only | 714 | 185/374 (49.47%) | 52/340 (15.29%) |
| B: Rejection-category coding | 714 | 189/374 (50.53%) | 60/340 (17.65%) |
| B: Recorded responses only | 664 | 189/350 (54.00%) | 34/314 (10.83%) |
| Combined: Rejection-category coding | 1428 | 374/748 (50.00%) | 112/680 (16.47%) |
| Combined: Recorded responses only | 1378 | 374/724 (51.66%) | 86/654 (13.15%) |

Note. All 50 unrecorded responses occurred in Case Series B: 26 for correct and 24 for incorrect recommendations. Case Series A had no unrecorded decisions. N in each percentage is the number of correct or incorrect recommendation observations included under the stated coding rule, not the total number of participants. The clinical-vignette sequence and correctness labels are identical in both summaries.

# Page-Submit Timing Audit

Timing fields were associated with clinical vignettes using the export question-block order: each analyzed R2T decision block was preceded by a Timing question containing First Click, Last Click, Page Submit, and Click Count fields. All 714 Case Series B observations had numeric Page Submit values. These are page-level elapsed times, explicit flags for automatic page advancement.

All unrecorded decisions had associated Page Submit values from 45.008 to 46.875 seconds, consistent with expiry-related nonresponse. The timing data therefore do not establish that all 50 observations resulted from exceeding the response limit.

Table S4. Case Series B page-submit timing by recorded decision status.

| **Decision status** | **Observations, n** | **Below 45 s, n** | **At least 45 s, n** | **Median s (range)** |
| --- | --- | --- | --- | --- |
| Unrecorded decision | 50 | 0 | 50 | 45.050 (21.909–46.875) |
| Recorded decision | 664 | 636 | 28 | 20.680 (1.678–52.302) |
| All Case Series B observations | 714 | 637 | 77 | 22.346 (1.678–52.302) |
